# Supplementary material for: Biophysical mechanisms of morphogenesis in lizard lungs
Source: bioRxiv. 2025 Sep 4:2025.09.01.673487. Preprint. [Version 1] doi: 10.1101/2025.09.01.673487 (PMC12424636; doi:10.1101/2025.09.01.673487)
Supplement: Supplement 1 [file NIHPP2025.09.01.673487v1-supplement-1.pdf]

bioRxiv preprint doi: https://doi.org/10.1101/2025.09.01.673487; this version posted September 4, 2025. The copyright holder for this preprint (which was not certified by peer review) is the author/funder, who has granted bioRxiv a license to display the preprint in perpetuity. It is made available under aCC-BY 4.0 International license.

## Supplemental Figures

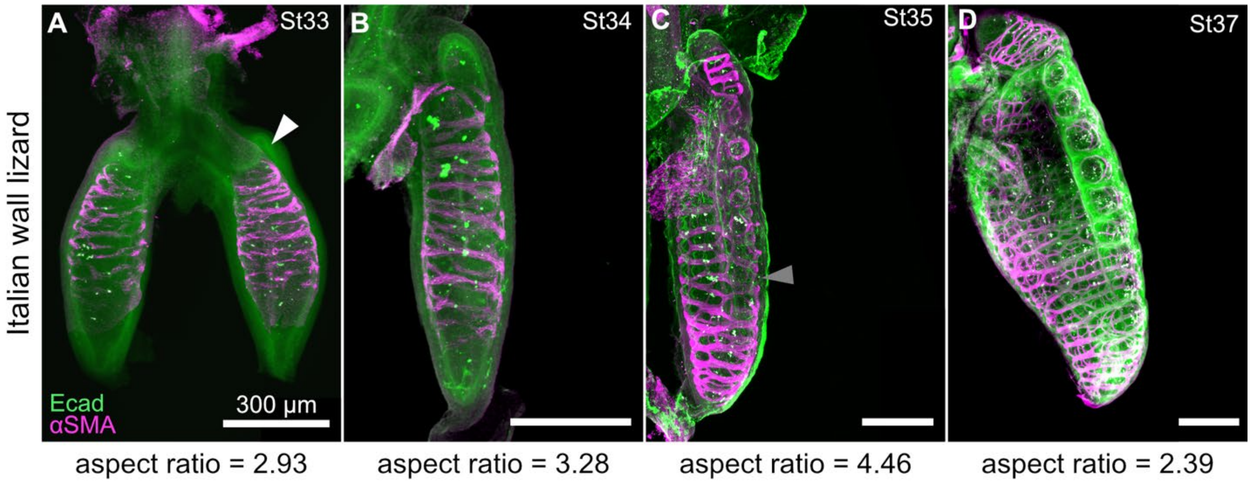


**Supplemental Figure 1. Opportunistic sampling, developmental staging, and aspect ratios of Italian wall lizard lungs.** Images show immunofluorescence for E-cadherin and aSMA (**A**–**D**). St, developmental stage, Scale bars, 300 μm. White arrow denotes emergence of the proximal sub-chamber. Gray arrows denote epithelium pushing through the smooth muscle mesh.

bioRxiv preprint doi: https://doi.org/10.1101/2025.09.01.673487; this version posted September 4, 2025. The copyright holder for this preprint (which was not certified by peer review) is the author/funder, who has granted bioRxiv a license to display the preprint in perpetuity. It is made available under aCC-BY 4.0 International license.

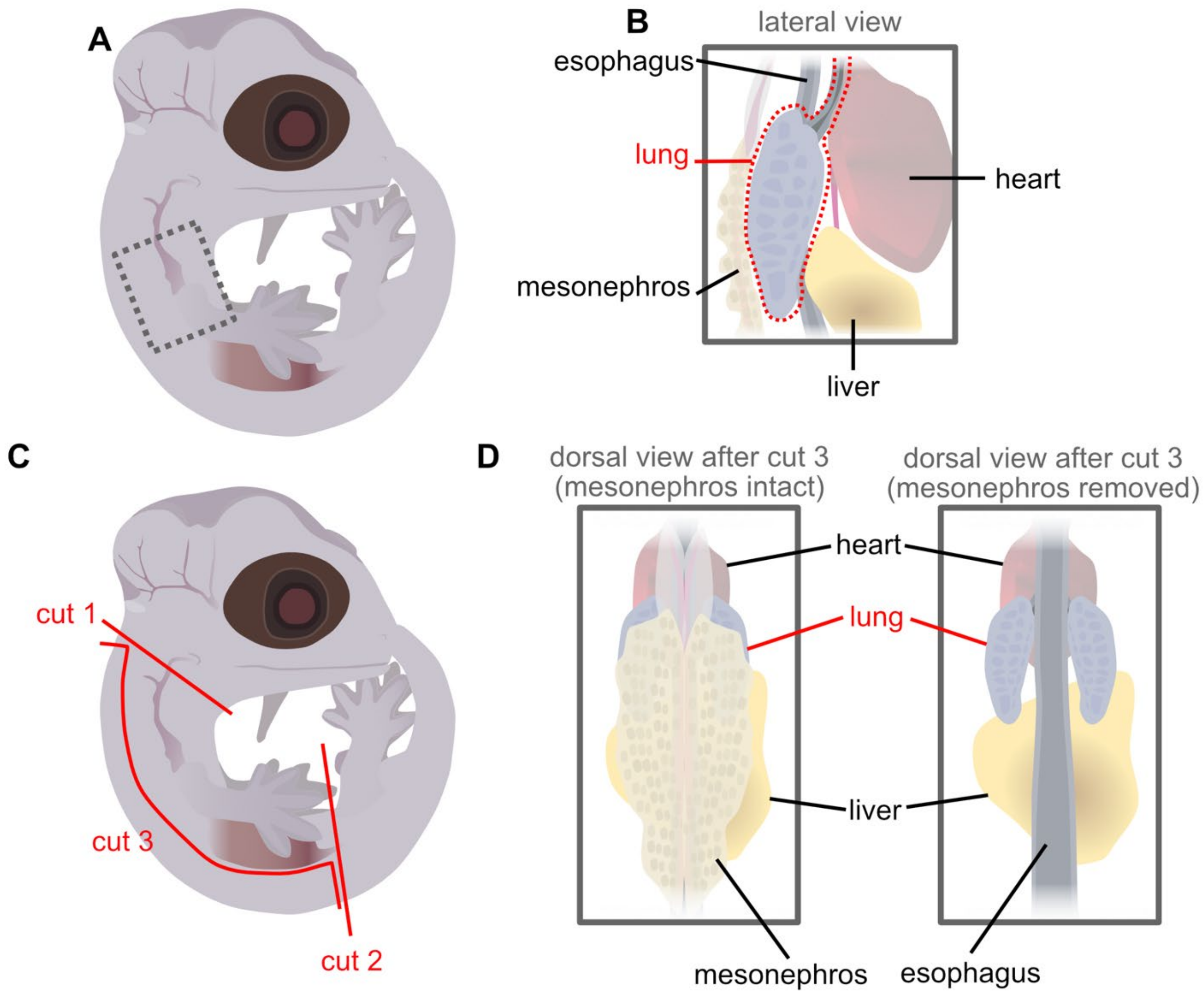


**Supplemental Figure 2. Schematic depicting procedure used to dissect lizard lungs**. **A**) Illustration of a lizard embryo in lateral view. Dashed box corresponds to the region of the torso where lungs reside. **B**) Illustration of the lateral view of lizard viscera. **C**) Illustration of a lizard embryo in lateral view depicting regions to cut with dissection forceps (red lines). **D**) Illustrations of lizard viscera in dorsal view. The lung is visible after removal of the mesonephros.
